# Differences in the clinical characteristics of COVID-19 patients who died in hospital during different phases of the pandemic: national data from Italy

**DOI:** 10.1007/s40520-020-01764-0

**Published:** 2020-12-21

**Authors:** Luigi Palmieri, Katie Palmer, Cinzia Lo Noce, Paola Meli, Marina Giuliano, Marco Floridia, Manuela Tamburo de Bella, Andrea Piccioli, Silvio Brusaferro, Graziano Onder, The Italian National Institute of Health COVID-19 Mortality Group (*), Luigi Palmieri, Elvira Agazio, Xanthi Andrianou, Pierfrancesco Barbariol, Antonino Bella, Stefania Bellino, Eva Benelli, Luigi Bertinato, Stefano Boros, Gianfranco Brambilla, Giovanni Calcagnini, Marco Canevelli, Maria Rita Castrucci, Federica Censi, Alessandra Ciervo, Elisa Colaizzo, Fortunato D’Ancona, Martina Del Manso, Corrado Di Benedetto, Chiara Donfrancesco, Massimo Fabiani, Francesco Facchiano, Antonietta Filia, Marco Floridia, Fabio Galati, Marina Giuliano, Tiziana Grisetti, Cecilia Guastadisegni, Yllka Kodra, Martin Langer, Ilaria Lega, Cinzia Lo Noce, Pietro Maiozzi, Fiorella Malchiodi Albedi, Valerio Manno, Margherita Martini, Alberto Mateo Urdiales, Eugenio Mattei, Claudia Meduri, Paola Meli, Giada Minelli, Manuela Nebuloni, Lorenza Nisticò, Marino Nonis, Graziano Onder, Lucia Palmisano, Nicola Petrosillo, Patrizio Pezzotti, Flavia Pricci, Ornella Punzo, Vincenzo Puro, Federica Quarata, Valeria Raparelli, Simone Rocchetto, Giovanni Rezza, Flavia Riccardo, Maria Cristina Rota, Paolo Salerno, Giulia Sarti, Debora Serra, Andrea Siddu, Paola Stefanelli, Manuela Tamburo De Bella, Dorina Tiple, Marco Toccaceli Blasi, Federica Trentin, Brigid Unim, Luana Vaianella, Nicola Vanacore, Maria Fenicia Vescio, Monica Vichi, Emanuele Rocco Villani, Amerigo Zona, Silvio Brusaferro

**Affiliations:** 1 Department of Cardiovascular, Endocrine-Metabolic Diseases and Aging, Istituto Superiore di Sanità, Rome, Italy; 2 Department of Geriatrics, Centro Medicina dell’Invecchiamento, Fondazione Policlinico A. Gemelli IRCCS, Rome, Italy; 3 National Center for Innovative Technologies in Public Health, Istituto Superiore di Sanità, Rome, Italy; 4 National Center for Global Health, Istituto Superiore di Sanità, Rome, Italy; 5 Department of Oncology and Molecular Medicine, Istituto Superiore di Sanità, Rome, Italy; 6 Office of the Director General, Istituto Superiore di Sanità, Rome, Italy; 7 Office of the President, Istituto Superiore di Sanità, Rome, Italy

**Keywords:** COVID-19, Older adults, Italy

## Abstract

**Background:**

Epidemiological data obtained during the initial wave of the COVID-19 epidemic showed that persons dying with COVID-19 were typically older men with multiple chronic conditions. No studies have assessed if the characteristics of patients dying with COVID-19 have changed in the second phase of the epidemic, when the initial wave subsided. The aim of the present study was to compare characteristics of patients dying with COVID-19 in Italy in the first ‘peak’ phase of the epidemic and in its second phase.

**Methods:**

Medical charts of patients with COVID-19 who died while in hospital in Italy were reviewed to extract information on pre-existing comorbidities, in-hospital complications, and disease trajectories. The course of the epidemic was classified in two 3-month periods: March–May 2020 and June–August 2020.

**Findings:**

Overall, in the Italian population, 34,191 COVID-19 deaths occurred in March–May 2020 and 1,404 in June–August 2020. Patients dying in March–May were significantly younger (80.1 ± 10.6 vs. 82.8 ± 11.1 years, *p* < 0.001) and less frequently female (41.9% vs. 61.8%, *p* < 0.001) than those dying in June–August. The medical charts of 3533 patients who died with PCR-confirmed SARS-CoV-2 infection in March–May 2020 (10.3% of all deaths occurring in this period) and 203 patients who died in June–August 2020 (14.5% of all deaths occurring in this period) were analysed. Patients who died in March–May 2020, compared to those who died in June–August 2020, had *significantly lower rates* of multiple comorbidities (3 or more comorbidities: 61.8% vs 74.5%, *p* = 0.001) and superinfections (15.2% vs. 52.5%, *p* < 0.001). Treatment patterns also substantially differed in the two study periods, with patients dying in March–May 2020 being less likely to be treated with steroids (41.7% vs. 69.3%, *p* < 0.001) and more likely to receive antivirals (59.3% vs. 41.4%, *p* < 0.001). Survival time also largely differed, with patients dying in March–May 2020 showing a shorter time from symptoms onset to death (mean interval: 15.0 vs. 46.6 days, *p* < 0.001). The differences observed between the two periods remained significant in a multivariate analysis.

**Interpretation:**

The clinical characteristics of patients dying with COVID-19 in Italy, their treatment and symptom-to-death survival time have significantly changed overtime. This is probably due to an improved organization and delivery of care and to a better knowledge of disease treatment.

**Supplementary Information:**

The online version contains supplementary material available at 10.1007/s40520-020-01764-0.

## Introduction

In Italy, the first case of COVID-19 was diagnosed on February 20, 2020 [1, 2], with the first related death occurring on February 21. The first phase of the epidemic reached a peak in Italy between March and April 2020. Following a 2-month lockdown period, the number of new cases and deaths was largely reduced in a second phase during the summer period 2020 [3].

The decrement in the number of cases in this second phase of the epidemic has reduced the burden and workload faced by emergency rooms, hospitals, and intensive care units, which were hugely under pressure during the peak phase [4]. This reduction in case numbers was also accompanied by a better knowledge about the clinical presentation of COVID-19, radiological findings, prognostic risk factors, and options for drug therapy, with potential improvements in management of the disease, accuracy of the diagnosis, treatment administered, and ultimately in survival.

Epidemiological data obtained in Italy at the beginning of the epidemic indicated that persons dying with COVID-19 were typically older men with multiple chronic conditions [5, 6]. Pneumonia and Acute Respiratory Distress Syndrome were observed in most COVID-19 patients who died, but non-respiratory complications (i.e. superinfections, acute renal failure, and cardiac injury) were also commonly diagnosed. However, until now, no studies have assessed whether the characteristics of patients dying with COVID-19 have changed in the second phase of the epidemic as a consequence of different disease management and delivery of care. The aim of the present study was to compare COVID-19 patients who died in Italy during the first (‘peak’, March–May 2020) and the second (June–August 2020) phase of the epidemic, evaluating in these two groups clinical characteristics (e.g., age, sex, comorbidities), in-hospital complications (e.g., superinfections), treatments administered (e.g., antivirals and steroids), and disease trajectories (e.g., survival time since symptom onset).

## Methods

### National COVID-19 surveillance system

At the outset of the COVID-19 outbreak, the Italian National Institute of Health (Istituto Superiore di Sanità [ISS]) established an integrated national surveillance system to collect information on all individuals with COVID-19 throughout the country. Data on all confirmed COVID-19 cases were obtained from all 19 Italian Regions and the two autonomous provinces of Trento and Bozen [2].

### Identification of COVID-19 deaths and clinical factors

All deaths occurring in patients who tested positive for SARS-CoV-2 through Reverse Transcription Polymerase Chain Reaction, independently from preexisting diseases that may have caused or contributed to death, are tracked by the surveillance system. To collect detailed clinical data, regions and autonomous provinces are asked to send to the ISS the medical charts and death certificates of SARS-CoV-2-positive patients who died in hospital. Clinical charts are then reviewed by a group of medical doctors at ISS. In this review, data on demographics, dates of hospitalization, SARS-CoV-2 testing, preexisting comorbidities, pharmacological treatments, date and cause of death, and complications during hospitalization are extracted from clinical records, and time from symptom onset to death is calculated [6].

We first considered for the present analysis all deaths occurring in SARS-CoV-2-positive patients reported in Italy to the surveillance system as of August 31, 2020 [7] and, within this group, all the cases with in-hospital death and complete medical records (including medical charts and death certificates) sent to the ISS. To analyze a homogeneous sample of patients in which COVID-19 was the main cause of death, we excluded from the analysis patients for whom COVID-19 or COVID-19-related conditions (i.e. pneumonia) were not listed in Part 1 of the death certificates, where the events leading to death, from the condition that initiated the chain of events to the subsequent conditions leading to death, are reported in causal sequence. We used this approach to ensure that the analysis did not include patients who had tested positive for SARS-CoV-2 but died as a result of another condition (for example trauma or cancer).

Periods of death were classified based on the course of the epidemic and number of deaths in Italy (Figure S1). Two 3-month periods were defined: March–May 2020 (“peak” phase) and June–August 2020.

### Statistical analyses

Age and sex of patients with complete medical records analyzed for this study significantly differed from what was observed in the whole population of patients dying with COVID-19 in the country (Table S1 and S2). The difference can be due to the fact that we examined cases of patients dying in-hospital and older patients might be more likely to die in other settings (i.e. nursing homes), particularly in the first phase of the epidemic [8]. However, to account for this age and sex difference, we standardized all means and prevalences by age and sex (including number and type of comorbidities, non-respiratory complications, treatments, days since symptom onset). Data presented for age and sex, however, are based on the 35,595 population (i.e., all COVID-19 deaths in Italy until the end of August 2020).

Characteristics of patients according to period of death were compared using *ANOVA* for continuous variables (i.e., age and days since symptom onset) and Fisher’s Exact Test for categorical variables (i.e., sex, comorbidities, non-respiratory complications, treatments). Binary logistic regression was used to identify factors independently associated with period of death. Variables included in this model were those associated with period of death at a level of significance ≤ 0.05 in univariate analysis. Analyses were performed using IBM SPSS Statistics 26 for Windows.

### Ethical Issues

On February 27, 2020, the Italian Presidency of the Council of Ministers authorized the collection and scientific dissemination of data related to COVID-19 by the ISS and other public health institutions [9].

## Results

As of August 31, 2020, a total of 35,595 deaths occurring in SARS-CoV-2-positive patients were reported in Italy to the national surveillance system [7], and 3945 (11.1%) complete medical records (including medical charts and death certificates) of patients who died in the hospital were examined in detail at the ISS. To analyze a homogeneous sample of patients in which COVID-19 was the main cause of death, we excluded from this group 209 patients (5.3%) for whom COVID-19 or COVID-19-related conditions (i.e. pneumonia) were not listed in Part 1 of the death certificates.

Overall, in the Italian population, 34,191 COVID-19 deaths occurred in March–May 2020 and 1404 in June–August 2020. As shown in Table 1, patients who died in March–May 2020 *had a significantly different age* (mean = 80.1 ± 10.6 vs. 82.8 ± 11.1, *p* < 0.001) and were less likely to be female, than those dying in June–August 2020 (41.9% vs. 61.8%, *p* < 0.001).

**Table 1:** Characteristics of COVID-19-related deaths according to study period

	All COVID-19-related deaths (*n* = 35,595)	March–May 2020 (*n* = 34,191)	June–August 2020 (*n* = 1404)	*p *value*
Demographics				
Age (years), mean ± SD	80.2 ± 10.6	80.1 ± 10.6	82.8 ± 11.1	< 0.001
Female, *n* (%)	15,187 (42.7)	14,319 (41.9)	868 (61.8)	< 0.001

	Whole study sample^a^ (*n* = 3736)	March–May 2020 (*n* = 3533)	June August 2020 (*n* = 203)	*p *value*
Demographics				
Age (years), mean ± SD	78.1 ± 11.5	77.9 ± 11.5	81.0 ± 11.2	< 0.001
Female, *n* (%)	1337 (35.8)	1231 (34.8)	106 (52.2)	< 0.001
N of comorbidities, *n* (%)^b^				
0	147 (4.0)	146 (3.9)	1 (0.5)	0.001
1	501 (13.7)	483 (13.3)	18 (10.5)	
2	764 (20.9)	737 (21.0)	27 (14.5)	
3 or more	2250 (61.4)	2095 (61.8)	155 (74.5)	
Non-respiratory complications^b^				
Acute renal failure, *n* (%)	827 (23.1)	773 (22.3)	54 (28.1)	0.045
Acute cardiac injury, *n* (%)	374 (10.4)	359 (10.3)	15 (8.2)	0.291
Superinfection, *n* (%)	634 (17.7)	535 (15.2)	99 (52.5)	< 0.001
Treatments^b^				
Antibiotics, *n* (%)	3167 (87.4)	2987 (87.0)	180 (90.9)	0.103
Antivirals, *n* (%)^c^	2170 (59.9)	2090 (59.3)	80 (41.4)	< 0.001
Steroids, *n* (%)	1571 (43.3)	1437 (41.7)	134 (69.3)	< 0.001
Disease trajectory^b^				
Time from symptom onset to death, mean ± SD	16.4 ± 14.7	15.0 ± 11.4	46.6 ± 28.8	< 0.001
Time from symptom onset to SARS-CoV-2 testing, mean ± SD	6.9 ± 7.3	6.9 ± 7.0	7.3 ± 10.1	0.533
From symptom onset to hospitalization, mean ± SD	6.0 ± 6.4	5.9 ± 5.9	8.0 ± 11.5	0.118

*SD *standard deviation

^a^Sample whose medical records were checked in detail by ISS

^b^Means and prevalence of variables in the two periods are age and sex standardized

^c^Including hydroxychloroquine

*p value for difference between study periods

Complete clinical records were available for 3533 cases of COVID-19-related deaths occurring in March–May 2020 (10.3% all deaths occurring in March–May 2020) and 203 deaths occurring in June–August 2020 (14.5%, all deaths occurring in June–August 2020). Table 1 summarizes the characteristics of the study sample by period of death. Differences in age and gender observed in the whole sample were confirmed in patients with complete clinical records (Table 1).

After age and sex standardization of the variables obtained after evaluating medical records, significant differences in number of pre-existing comorbidities and non-respiratory complications was observed between the two study periods, with patients who died in the June–August 2020 period presenting with more pre-existing comorbidities and non-respiratory complications, including acute renal failure and superinfections than those who died in the first phase (Table 1). Similarly, treatment patterns significantly differed; patients who died in the June–August 2020 period were more likely to be treated with steroids, and less likely to receive antivirals compared to those who died in the first phase. Finally, patients who died in the first phase (March–May 2020) period had a shorter time from symptom onset to death.

**Fig. 1:**
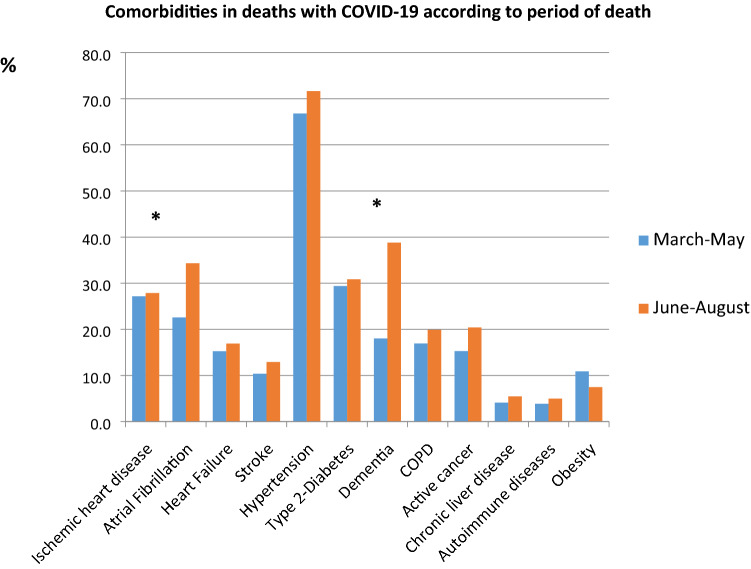
Most common pre-existing comorbidities by study period. Prevalences are age and sex standardized. *p value for difference between study periods < 0.001

Figure 1 shows most common comorbidities observed in patients who died over the two phases of the pandemic. With the exception of obesity, all other conditions were more frequent in patients who died in the June–August period, although a significant difference was only found for atrial fibrillation (22.6% vs. 34.3%, *p* =  < 0.001) and dementia (18.0% vs. 38.8%, *p* =  < 0.001).

Table 2 reports results of the multivariate analyses identifying variables independently associated with death occurring in the June–August period. Overall, patients who died in this second phase were significantly older, more frequently female, had a higher number of pre-existing comorbidities, and a higher probability of superinfections. In addition, patients who died during the June–August period were less likely to receive antiviral drugs, more likely to be treated with steroids, and they had a significantly longer time from symptom onset to death than those dying in the March–May 2020 period.

**Table 2:** Multivariate analysis. Factors independently associated with death occurring June–August 2020 compared to March–May 2020

Variable	AOR	95% Confidence Interval
Demographics		
Age (per 1-year increment)	1.050	1.027–1.073
Female	1.810	1.186–2.760
Number of comorbidities^a^		
0 or 1	Ref	–
2	1.467	0.638–3.379
3 or more	2.233	1.087–4.586
Non-respiratory complications		
Acute renal failure	0.986	0.613–1.585
Superinfection	2.740	1.723–4.356
Treatments		
Antivirals^b^	0.307	0.196–0.479
Steroids	2.335	1.485–3.670
Disease trajectory		
Days from symptoms onset to death (per 1-day increment)	1.065	1.054–1.076

*AOR* Adjusted Odds Ratio

^a^0 and 1 categories were merged due to the limited number of cases with 0 comorbidities in the June–August period (*n* = 1)

^b^Including hydroxychloroquine

## Discussion

In the present study, we show that characteristics of patients who died with COVID-19 in Italy have largely changed over time. Patients who died in the second phase of the epidemic were older, more likely to be women, and had a higher probability of superinfections, a larger comorbidity burden, and longer survival from symptom onset compared to people who died in the first phase (March–May 2020). In addition, the treatment approach has changed over the two periods as those who died in the second phase were less likely to receive antivirals and more likely to be treated with steroids.

These findings might be explained by different factors. First, there was less burden on the healthcare system in the second phase of the epidemic [4]. In the peak first phase, emergency rooms, hospitals, and intensive care units were challenged by the need to simultaneously provide care to a high number of critically ill patients [10]. Second, the organization of care improved in the second phase of the epidemic. COVID-19 and non-COVID-19 streams of care were created in the hospitals, community-care approaches were implemented, specific diagnostic and therapeutic pathways were carried out [11]. Finally, knowledge on COVID-19 diagnosis and treatment has improved over time, potentially leading to more accurate diagnosis and better treatment [12]. This is also confirmed by the change in treatment patterns between the two study periods: during the early phase, more patients were treated with antivirals, which research has now shown to be less effective than originally hoped [13]. Similarly, use of steroids increased in the second phase after this drug was proven effective in reducing COVID-19 complications [14]. All these factors may have improved survival in COVID-19 patients and led to a shift of mortality toward older, more vulnerable and complex patients.

Interestingly, superinfections were more commonly observed in patients dying in the second phase of the epidemic. This might be due to the fact that, in this latter period, typical COVID-19 respiratory conditions were better treated and managed so death may have occurred when patients experienced additional non-respiratory complications that further worsened health status, leading to a negative prognosis. In addition, the increase in use of steroids in the second phase may have facilitated the onset of superinfections.

In the first phase of the epidemic, there was a larger proportion of men among patients who died with COVID-19 than the second phase. This finding is difficult to explain. Several studies have underlined that men have a higher risk of COVID-19-related mortality than women, and sex differences in clinical manifestations and transitions of care have been described [15, 16]. In the Italian population, the time from symptom onset to hospital admission and diagnostic testing were slightly longer in men and this might have explained differences in prognosis in the first phase of the epidemic [16]. The improvement in testing ability and the decreased burden on hospital may have flattened these differences and reduced the mortality gap in the second phase.

Our findings should be interpreted in light of potential limitations. First, they focused only on patients who died in hospital, while deaths occurring at home or in long-term care facilities were not included. Particularly in the first phase of the epidemic, hospitals were overwhelmed and oldest and most severely impaired patients were neither tested, nor transferred and died at home or in long-term care facilities [10]. However, the data are still informative for comparing clinical history and disease progression among the subset of hospitalized patients. Second, we only reviewed the charts of patients who died; data on hospitalized patients who survived were not collected. Thus, we were unable to assess if the case fatality rate has changed in the second phase of the epidemic, although until widespread effective testing and screening procedures are in place (including testing for asymptomatic cases), these estimates are unlikely to be reliable. This also means that our data cannot determine risk factors for mortality; rather we only describe how the characteristics of the patients who have died have changed. Finally, the generalizability of our findings might be limited as we provide data only on Italian patients, although the data come from all Regions of Italy, thus providing a comprehensive picture of the situation in one country.

In conclusion, we show that characteristics of patients in Italy who died with COVID-19, their treatment, and disease trajectory have changed over time. This is probably due to an improvement in organization of care delivery and in knowledge of disease treatment. Future studies are needed to confirm this finding in other countries and over longer time periods.

## Supplementary Information

Below is the link to the electronic supplementary material.

Supplementary file1 (DOCX 21 KB)
