# Supplementary material for: Differences in the clinical characteristics of COVID-19 patients who died in hospital during different phases of the pandemic: national data from Italy
Source: Aging Clin Exp Res. 2020 Dec 21;33(1):193–9. doi: 10.1007/s40520-020-01764-0 (PMC7750107; doi:10.1007/s40520-020-01764-0)
Supplement: Supplementary file 1 — Supplementary file1 (DOCX 21 KB) [file 40520_2020_1764_MOESM1_ESM.docx]

**Figure S1.** Number of SARS-CoV-2 positive patients who died in Italy between March and August 2020

**Table S1.** Sample representativeness

|  | **March-May 2020** | | | | |
| --- | --- | --- | --- | --- | --- |
|  | All deaths,  n | % | Study sample, n | % | *p-value* |
| **Sample size** | 34,191 | 100% | 3,533 | 100% | *-* |
| **Age** |  |  |  |  | *<0.001* |
| < 60 | 1,580 | 4.6% | 273 | 7.7% |  |
| 60-69 | 3,478 | 10.2% | 418 | 11.8% |  |
| 70-79 | 9,045 | 26.5% | 992 | 28.1% |  |
| 80+ | 20,088 | 58.8% | 1,850 | 52.4% |  |
| **Women** | 14,319 | 41.9% | 1,231 | 34.8% | *<0.001* |
|  | **June-August 2020** | | | | |
| **Sample size** | 1,404 | 100% | 203 | 100% | *-* |
| **Age** |  |  |  |  | *0.451* |
| < 60 | 66 | 4.7% | 13 | 6.4% |  |
| 60-69 | 94 | 6.7% | 18 | 8.9% |  |
| 70-79 | 248 | 17.7% | 36 | 17.7% |  |
| 80+ | 996 | 70.9% | 136 | 67.0% |  |
| **Women** | 868 | 61.8% | 106 | 52.2% | *0.009* |

**Table S2.** Comparison of age and sex distribution of the sample in the 2 periods

|  | **March-May 2020** |  | **June August 2020** |  |  |
| --- | --- | --- | --- | --- | --- |
|  | Study sample, n | % | Study sample, n | % | *p-value* |
| **Sample size** | 3,533 | 100.0% | 203 | 100.0% |  |
| **Age** |  |  |  |  |  |
| < 60 | 273 | 7.7% | 13 | 6.4% | *0.491* |
| 60-69 | 418 | 11.8% | 18 | 8.9% | *0.201* |
| 70-79 | 992 | 28.1% | 36 | 17.7% | *0.001* |
| 80+ | 1,850 | 52.4% | 136 | 67.0% | *<0.001* |
| **Women** | 1,231 | 34.8% | 106 | 52.2% | *<0.001* |
